# Supplementary material for: Using an analogue-digital hybrid clinical data management platform during a two-dose preventive Ebola virus vaccine trial in Goma, the Democratic Republic of the Congo
Source: PLOS Glob Public Health. 2025 May 2;5(5):e0004487. doi: 10.1371/journal.pgph.0004487 (PMC12047755; doi:10.1371/journal.pgph.0004487)
Supplement: Attachment — Submitted filename: PLOS_Response_Reviewer_Comments_12DEC24.docx [file pgph.0004487.s002.docx]

**Reviewer 1**

Line 61: Please go into a little more detail on the types of chronic conditions that were collected. How could these conditions influence the attendance to the clinics for vaccination?

The question about ‘chronic’ health problems was kept intentionally broad to allow for participants to describe health issues which they felt were an important part of their health experience. This question was included in the trial design to establish whether there may be adverse events that associated with such conditions.

The participants of the trial reported a broad range of conditions, which can be summarised as below and have been added to the results section of the manuscript.

1. **Infections**, including STIs, Tuberculosis, Herpes and others
2. **Musculoskeletal and Joint Issues**, especially arthropathies, rheumatoid arthritis, fractures and soft tissue injuries
3. **Gastrointestinal Issues**, including IBD/IBS, gastric issues and ulcers
4. **Neurological and Mental Health Conditions** including Schizophrenia, Bipolar disorder, epilepsy, migraine and seizures
5. **Cardiovascular and Respiratory Issues**, including hypertension, diabetes, heart disease, asthma and pneumonia-like symptoms
6. **Skin and Allergy Conditions** such as psoriasis, eczema and allergies.
7. **Trauma and Substance-Related Issues** including burn injuries, substance abuse and narcotic addiction

Please note that some nuance may be lost in transcription (by enumerators) and translation (of French text to English).

For the purposes of this study, we used these questions as a generalised indicator of a person’s perception of their own health at the times they attended our study sites. In keeping with our previous research on self-perception of risk^[1]^ we anticipated that a generalised perception of personal risk might be an influencing factor when seeking vaccination against a potentially high consequence epidemic.

Line 178-179: Could you elaborate briefly why the pregnancy test was optional or why it is relevant information for this paper?

The pregnancy test was optional as it would not have been ethical to enforce pregnancy testing, particularly as there is no evidence that the vaccines are teratogenic. The use of pregnancy testing is important for this study as a subset of pregnant women were actively followed-up for serious adverse events. They may therefore have been less likely to rely on the date of the Enhanced Participant Record Card (EPRC) to return for dose 2 as they received regular phone calls from the study team. Pregnancy and testing for pregnancy were also a factor which complicated the workflows and movements of participants between the various stations, as is shown in figure 4.

This has been added to the methods and further clarified in the discussion.

Line 358-361: Can you clarify which data was collected electronically and at what time point?

Open Data Kit (ODK) was used to collect data at each of the vaccination stations (consent and registration, eligibility, pregnancy testing, vaccination and the 15-minute observation period following vaccination). This is clearly indicated in figure 1 but we have also added this information to the methods text (lines 245-246).

Did the participant who received the second dose twice come to the vaccination site twice on the same day, and was the electronic data collection tool therefore not updated yet?

On review of our investigation forms, we noted that two participants were inadvertently vaccinated twice. The participants were not vaccinated on the same day, rather they attended the vaccination site some months after receiving the dose 2, claiming to have lost their vaccination cards. Unfortunately, at that timepoint, study staff did not verify their vaccination status and they received dose 2 for a second time. This was therefore an episode human error, rather than an error of the electronic data collection tool. The details have been clarified in lines 434-442.

Line 365: can you elaborate on what you mean by "eCRF deviated from the study protcol"?

We have now clarified that this type of deviation occurred when 26 eCRFs were completed even though they were not required by the protocol as opposed to the 23 participants for whom an eCRF was missing.

General: Is it possible to explain what data was collected in the eCRF? It is unclear whether more information than what was available on the perforated cards was collected, and if so, how and when this data became available on the central server.

Substantial data were collected for trial purposes on the electronic case report form (eCRF). The EPRC represented only that fraction of the database which had utility in identifying participants, assessing their vaccine history and directing them between stations during visits to the study sites. A summary of data collected in the eCRFs is given in Figure 3 and added to lines 249-251. Data were automatically synchronised to the server once WiFi was available, normally at the end of each day. This has been clarified in line 275.

**Reviewer 2**

1. The title of the paper is too long and contain an excessive explanation of the study. Research article titles should be very concise and communicate the theme of the study. Kindly reconstruct the title.

We agree with the reviewer that the title was too long. This has now been shortened to ‘Using an analogue-digital hybrid clinical data management platform during a two-dose preventive Ebola virus vaccine trial in Goma, the Democratic Republic of the Congo’.

2. The novelty of the study is not clearly captured in the introduction. I expect the authors to carry out an extensive literature study on the existing clinical data management system (CDMS). This will enable the reader to identify the gaps in the existing system and appreciate the contribution to literature for this study. Please carry out a good literature review and be clear with the novelty of the study.

There is limited background literature on which to draw, but we highlight the challenges of developing a clinical data management system (CDMS) in settings with limited IT infrastructure by providing detailed examples of the development of two other CDMS deployed during the West Africa Ebola outbreak in Sierra Leone and Guinea. Although our system has some similarities with these systems, we added the novel component of the ERPC and remote server synchronisations which are a key focus of the work presented here. This has been added to the introduction.

3. The authors should clearly state with reasons the statistical designs applied in this research. For instance, be clear with the research design, sampling technique and other salient techniques.

We have added the research design for the trial based on the protocol by Watson-Jones et al., 2022.^[2]^ Information about the study site for the trial and how participants were recruitment are already provided in our methods for this paper under review. There is no sampling technique *per se* for this study; rather we analysed data from those participants who received the second vaccine dose. We have highlighted this in the text. In the analysis section we have described the statistical method used and have now justified this with substantial detail. We have also amended the text to highlight why the covariates listed were included in the model, to ‘adjust for potential confounding.’

4. The arrangement format of the Figures in the study is inappropriate. You may create an appendix and send both the figures and their titles there or let them appear in the main work together. Titles for figures cannot appear in the work while the figures appear later.

We share the frustration of the reviewer that as per the journal’s requirements, the figures are submitted separately, while the titles appear in the main body of the text. This is beyond our control,but we fully expect that the final paper will be properly type-set.

5. The study results are quite questionable since I see no proper comparison with existing results. Authors are encouraged to compare their results to that of the existing approaches. This will help verify the validity of their results.

As this is a novel system within a niche discipline, there are few structured papers describing studies that focus specifically on methods for deploying new CDMS platforms. Consequently, the potential for direct comparisons with other studies is limited, particularly with regard to the technical implementation of an off-grid capable CDMS. It is also practically unfeasible to conduct side-by-side comparisons of two different CDMS systems while undertaking a regulated clinical trial in a conflict zone during an outbreak of a high-consequence infectious disease. This limitation reflects the challenging context in which such systems are deployed and highlights the lack of an established gold standard for off-grid CDMS, which was precisely the rationale for developing this system.

In terms of the analyses presented in Table 1, the within-study comparisons provide relative odds of demographic groups relying on the EPRC, which inherently require no external validation. Such comparisons are well-suited to the data and research question, and we believe the methods and findings to be both reasonable and appropriate.

We have attempted to contextualise our results with reference to existing studies where possible. For example, we included a comparison with a randominsed controlled trial (RCT) conducted in Kenya that found individuals who received an SMS and a phone call or an in-person reminder were 1.4 times as likely to return for repeat HIV testing compared with those who received only an appointment card^[3]^. Similarly, we added an example from a study in Tanzania^[4]^, where text message reminders showed no difference in follow-up attendance for cervical cancer screening compared to an appointment card. While these comparisons provide some broader context, their value is inherently limited because the devices, methods, and protocols used in these studies differ substantially from those used here, beyond the shared concept of using ‘appointment cards’ or similar devices.

6. The paper contains enormous grammatical errors. Please fix them.

We have reviewed the text throughout and we hope that we have corrected any outstanding grammatic errors.

7. I have challenge with the contribution of this research work, since the figures are poorly presented. I am unable to read the patterns in there to assess its feasibility.

We are unsure whether the reviewer’s point here relates to their dissatisfaction with the way the journal sent the manuscript and associated figures to them (see above), or if they have a wider problem with the figures themselves. We believe that our figures our clear and well described by the legends and defer to the editor to advise us further on this matter if any issue remains.

8. Authors may employ more statistical tools like graphs to present the results in the table. That will make reading quite interesting.

Given the relative simplicity of the statistical testing performed, we feel that it might be rather redundant to provide both the table (Table 1) and a chart of the same data in the form of a forest plot. In this case, whilst a chart might be clearly illustrative of the confidence intervals on the relative odds estimates, it would provide less specific information (i.e. counts and percentages) than is already presented in the table. We also note that the paper currently has five figures and just this one table.

9. The limitation of this current study is enormous and questions what problem exactly has been solved by this study.

We clearly articulate the problem being addressed in the introduction where we discuss the challenges of conducting clinical trials in settings with intermittent or non-existent internet and power connectivity, particularly in complex humanitarian contexts such as health emergencies. We also highlight the need for (and lack of) robust data management systems that function effectively in these off-grid conditions. The data and results we present, along with the coherent and complete data set collected during the trial, provide evidence that the hybrid database used in this study was an effective CDMS device for the logistical delivery of the trial. That the recall rate remained high (even against the backdrop of COVID-19) is a source of evidence that the device may also improve on other methods whilst quite uniquely being amenable to working mostly off-grid.

We fully acknowledge and discuss the various limitations of our study, some of which warrant further study and platform development, but we believe that none these, are ‘enormous’. Although not an objective of the study, we note that we were unable to directly compare the EPRC with other types of reminders for dose 2, which we acknowledge would have been an opportunistic analysis rather than a fundamental flaw.

We have also described potential improvements to the system, such as enabling the recording of contraindications away from study sites, enhancing the granularity of the EPRC, storing cards at vaccination sites, and cross-checking participant eligibility using registration books. However, we view these as opportunities for future development rather than critical shortcomings of the current work.

It is important to emphasize that we had to give some concession to the very challenging context in which this trial was conducted — a regulated clinical trial in a conflict zone during an outbreak of a high-consequence infectious disease. Similar clinical studies during the West Africa Ebola outbreak also faced limitations and provided recommendations for future systems. ^[5]^ ^[6]^ ^[7]^ ^[8]^ ^[9]^ For example, as already described in our discussion, Carter et al., 2018 struggled with internet connectivity and had to revert to using paper forms^5^ and in the introduction, the inability of Hossman et al., 2019 to validate their tool,^7^ We have also added to the discussion the inability of Jobanputra et al., 2017 to implement their electronic tool in time to determine whether there was any improvement in the efficiency of the Ebola Medical Centres (EMCs).^6^ In this light, we believe that the limitations we describe are reasonable and expected, and that the study represents a significant step forward in addressing the lack of off-grid-capable CDMS solutions.

10. Authors should work on the in-text citations. Check the journal referencing formats and follow appropriately.

We noticed that the last few references had not been added to the bibliography. We apologise for this and have amended the paper so that these appear properly.

1. Rogers NT, Waterlow NR, Brindle H, Enria L, Eggo RM, Lees S, Roberts CH. Behavioral Change Towards Reduced Intensity Physical Activity Is Disproportionately Prevalent Among Adults With Serious Health Issues or Self-Perception of High Risk During the UK COVID-19 Lockdown. Front Public Health. 2020 Sep 30;8:575091.
2. Watson-Jones D, Kavunga-Membo H, Grais RF, Ahuka S, Roberts N, Edmunds WJ, Choi EM, Roberts CH, Edwards T, Camacho A, Lees S, Leyssen M, Spiessens B, Luhn K, Douoguih M, Hatchett R, Bausch DG, Muyembe JJ; DRC-EB-001 protocol writing team. Protocol for a phase 3 trial to evaluate the effectiveness and safety of a heterologous, two-dose vaccine for Ebola virus disease in the Democratic Republic of the Congo. BMJ Open. 2022 Mar 8;12(3):e055596. doi: 10.1136/bmjopen-2021-055596. PMID: 35260458; PMCID: PMC8905941.
3. Mugo PM, Wahome EW, Gichuru EN, Mwashigadi GM, Thiong’o AN, Prins HAB, et al. Effect of Text Message, Phone Call, and In-Person Appointment Reminders on Uptake of Repeat HIV Testing among Outpatients Screened for Acute HIV Infection in Kenya: A Randomized Controlled Trial. PLOS ONE. 2016 Apr 14;11(4):e0153612.
4. Linde DS, Andersen MS, Mwaiselage J, Manongi R, Kjaer SK, Rasch V. Effectiveness of One-Way Text Messaging on Attendance to Follow-Up Cervical Cancer Screening Among Human Papillomavirus–Positive Tanzanian Women (Connected2Care): Parallel-Group Randomized Controlled Trial. Journal of Medical Internet Research. 2020 Apr 2;22(4):e15863.
5. Carter RJ, Idriss A, Widdowson MA, Samai M, Schrag SJ, Legardy-Williams JK, et al. Implementing a Multisite Clinical Trial in the Midst of an Ebola Outbreak: Lessons Learned From the Sierra Leone Trial to Introduce a Vaccine Against Ebola. J Infect Dis. 2018 May 18;217(suppl_1):S16–23.
6. Jobanputra K, Greig J, Shankar G, Perakslis E, Kremer R, Achar J, et al. Electronic medical records in humanitarian emergencies – the development of an Ebola clinical information and patient management system. F1000Res. 2017 Feb 23;5:1477.
7. Hossmann S, Haynes AG, Spoerri A, Diatta ID, Aboubacar B, Egger M, et al. Data management of clinical trials during an outbreak of Ebola virus disease. Vaccine. 2019 Nov 15;37(48):7183–9.
8. Larivière Y, Matuvanga TZ, Lemey G, Osang’ir BI, Vermeiren PP, Milolo S, et al. Conducting an Ebola vaccine trial in a remote area of the Democratic Republic of the Congo: Challenges, mitigations, and lessons learned. Vaccine. 2023 Dec 12;41(51):7587–97.
9. Oza S, Jazayeri D, Teich JM, Ball E, Nankubuge PA, Rwebembera J, Wing K, Sesay AA, Kanter AS, Ramos GD, Walton D, Cummings R, Checchi F, Fraser HS
   Development and Deployment of the OpenMRS-Ebola Electronic Health Record System for an Ebola Treatment Center in Sierra Leone
   J Med Internet Res 2017;19(8):e294
