# Supplementary material for: Using an analogue-digital hybrid clinical data management platform during a two-dose preventive Ebola virus vaccine trial in Goma, the Democratic Republic of the Congo
Source: PLOS Glob Public Health. 2025 May 2;5(5):e0004487. doi: 10.1371/journal.pgph.0004487 (PMC12047755; doi:10.1371/journal.pgph.0004487)
Supplement: Attachment — Submitted filename: Reviewer_Comments_15MAR25.docx [file pgph.0004487.s003.docx]

Reviewer #1: 1. Thank you for adding the pregnancy clarifcation, the reasoning is now clear. Do I understand correctly that pregnancy was thus not an exclusion criteria for vaccination? I believe some punctuation is missing in the added text. If this is corrected, I have no further comments on this point. Please also be consistent in refering to the doses: Dose one versus Dose 2 in one sentence. Propose to write it out in text or in letters but not the combination.

Thank you for your query. Yes, you understood correctly that pregnancy was not a reason for exclusion from vaccination. We have added a comment in lines 216 to 217 to clarify this.

Reviewer #2: I have gone through the revised manuscript and find he results to be interesting. I have some few comments which will improve the paper.

1. The abstract should be a paragraph and should be concise.

The abstract has been converted to one paragraph and the text reduced. The word count is now 259.

2. There are still some errors in the manuscript, for instance, check line 67 on page 2.

The typing error on page 2 has been amended. The grammar has also been checked again throughout and amended accordingly.

3. The conclusion is not accurately written. key observations from the research were not reported. Also, it fails to state the weakness of the research and also recommendations for future.

We acknowledge your concerns with the conclusion and therefore have amended this to include key findings including EPRC retention and use as a prompt to return for dose 2. We also included important limitations such as the inability of the EPRC to record events occurring in locations other than the vaccination site and the lack of a robust comparative analysis of the EPRC with other prompts to return for dose 2. We have therefore also included recommendations for these.

4. The figure titles are too broad. I suggest that concise titles should be given, and the details should be given under the discussion of results.

We have reduced the text for the figure titles and added the remaining original text as legends.
